# "The Hospital" Nursing Mirror

**Published:** 1900-07-14

**Authors:** 


*The Hospital*, July 14, 1900.

### "The Hospital" Nursing Mirror.

Being the Nursing Section of "The Hospital."

[Contributions for this Section of "The Hospital" should be addressed to the Editor, The Hospital, 28 & 29, Southampton Street, Strand, London, W.C., and should have the word "Nursing" plainly written in left-hand top corner of the envelope.]

### Notes on News from the Nursing World.

### NURSES FOR CHINA.

The announcement that the Government have accepted the offer of the Executive Committee of the hospital ship "Maine" for service in China is satisfactory. Whatever excuses for the deficiency of nurses in South Africa may be urged, there can be none for not recognising the immediate necessity of sending out a supply of nurses to the base hospitals which are required in the Far East. It may be practicable to spare a certain number from India; and possibly some American nurses, who can reach Shanghai from San Francisco in a little over a fortnight, may be available. But we must mainly rely upon our resources at home.

### THE WAR OFFICE AND NURSING AT THE FRONT.

The Commission of Inquiry into the charges of Mr. Burdett-Coutts, M.P., cannot be an entirely satisfactory body unless it has two good men of business upon it. From two very different sources comes strong confirmation of the accusations of lack of nursing. In an address delivered last week at St. George's Hospital, Mr. Clinton T. Dent said :—

"The description published of the nursing at Bloemfontein had come as a rude shock to many. The events to which attention had recently been drawn by Mr. Burdett-Coutts took place after he (Mr. Dent) had left South Africa, and in towns which he had not visited, but from what he had seen he had formed the opinion that, while the hospitals in South Africa were most distinctly good, they ought to have been 50 per cent. better. A base hospital, properly speaking, contained 520 beds, of which 20 were for officers, but this number might be increased indefinitely. Nine nursing sisters were allotted to the 520 beds, somewhere about one nurse for every 65 patients. At St. George's Hospital the proportion was one to three. The number of orderlies allowed was 78 for a base hospital, and if these were added to the nurses the proportion of nursing attendants was about one to five. The orderlies, however, had to work as porters, as wardmaids, and on transport work. There were now about 600 nurses engaged in this service; but there were 18,000 beds to be nursed in South Africa, and nurses should be sent out by thousands."

The Hon. A. Stanley, M.P., who returned from the front on Saturday evening, is equally emphatic in his testimony. "Speaking roughly," he observed, "the whole requirements for sick and wounded were underestimated from the very beginning. Even when the number of sick, not so much wounded as enteric cases, grew to such enormous proportions, the authorities did not appear to appreciate what they had to cope with. Whose was the fault was a matter for investigation, and he was very glad to see that a commission was appointed. As far as he could see, the system on which the R.A.M.C. worked was bad, but no praise could be too great for individual doctors, nurses, and trained orderlies, who were working themselves to death in gallant efforts to perform an almost impossible task. It was not uncommon for a staff calculated to look after 500 patients to have to attend to 1,200 or 1,500."

### THE ORDERLIES AND THE NURSING OF TYPHOID.

In a letter written to a Sister at Adelaide, which is published in the *Adelaide Observer* of June 2nd, Miss M. S. Bidmead, one of the nurses who went to South Africa from Western Australia, and is stationed at the General Hospital, Wynberg, says:—

"The question of the 'orderly' system is a grave one. We have only been here three weeks, but that is enough to show us that there are serious disadvantages in it. Sister Hardament, the superintendent sister, says their best orderlies are not here, and for the credit of the St. John Ambulance and R.A.M.C. societies I should sincerely hope they are not. These trained orderlies do them great discredit, though I understand that I have been rather unfortunate in those I have had. One volunteer, who has never been trained, but who is willing to carry out your orders, and does not give himself airs, is worth ever so many of the trained ones. Sometimes I have a 'relief' orderly, just for two hours—I had one to-night—who gives one an idea of what a treasure a really good orderly must be. But they are few and far between; the first-class men have all been sent on to the front. Their ways of nursing enteric are marvellous—that is the only word for it. I am sure your cap would stand upright, and your hair, too. Mine nearly did at first. Imagine an enormously thick blanket underneath the sheet on which they lie—the enteric patient I mean; it is considered anything but correct to call them typhoid—no mackintosh, a thick sheet, another heavy military blanket, and a monster quilt above all. Then they wear a flannel shirt and a thick twilled one as well! And some of them with temperatures of 105 and 106 degrees."

On the other hand, Dr. Conan Doyle, writing to the *British Medical Journal*, pays a striking tribute to the devotion of the orderlies, of whom he says, "They are patriots, these men; for many of them have accepted a smaller wage in order to take on these arduous duties, and they are facing danger for 12 hours of the 24, just as real, and much more repulsive, than the scout who rides up to the strange kopje, or the gunner who stands to his gun with a pom-pom quacking at him from the hill." Mrs. Bagot, wife of Captain Bagot, M.P., who has just returned from South Africa, observed on Saturday that she had no word strong enough to express her respect and admiration for the Army Nursing Sisters, and for all these men in the Medical Corps, "and particularly the orderlies." There are orderlies *and* orderlies.

### CHILDREN ON BOARD A BOAT WITH TYPHOID PATIENTS.

There seems to be no reason why private persons with families of children should have been allowed to travel on the s.s. "Bavaria," with its load of 1,100 enteric patients and convalescents from South Africa. One of the nurses in charge informs us that the steamer carried twenty children, and that among them was a boy who had been sent away from school on account of an outbreak of measles. He had been seen by a doctor, and pronounced free from infection; nevertheless, he developed measles during the voyage, the other children caught it from him, and, more serious still, one of the nurses in charge of the soldiers also developed the complaint. This nurse is now in London, but is greatly inconvenienced by having to go into small private lodgings, instead of joining a friend (also a nurse on board the "Bavaria") at the hotel. Fortunately, none of the patients caught the complaint, though they might, of course, easily have done so. "If they had been surgical cases it would not have mattered so much," said the nurse; "but it is essential that typhoid convalescents should have peace and quietness, and it was very bad for them, besides the risk of infection." No wonder the officer in charge was —to use a mild term—annoyed.

### NURSING AT MARITZBURG.

An Army Reserve Sister, writing from Maritzburg, says: "This place is invaded with dogs and cats. The other night I found that a frisky young dog had walked off with my one and only sponge, and had literally worried it to atoms. It was no use trying to scold the animal; he lay on his back, wagged his tail, and looked at me with such a pathetic look in his great eyes that in spite of real personal discomfort I was compelled to seal my pardon with a pat. Within the last fortnight all my good cases have been well enough to go back to England, and we are fairly quiet here. I have only one patient who causes me any anxiety—a typhoid, whose temperature will not come down. My vanity is now suffering a severe blow. My hair is nearly all gone, and I have only enough for one wee plait. Long may that remain. Our sisters at the Cape write in the best of spirits; they live at the foot of the Table Mountain. Being winter, the mornings and evenings are cold, but the middle of the day is quite as warm as a summer day at home."

### PROVISION FOR INVALIDED NURSES FROM THE FRONT.

The need of provision for invalided nurses who have suffered in the discharge of their duties in South Africa does not require to be insisted upon. The only question is the extent and kind of the provision. Mr. Fripp mentions in a letter to the *Guy's Hospital Gazette* one of the sisters who "has been ill ever since she left England"; but several have fallen, and will continue to fall, victims either to typhoid or to overwork. It is therefore a matter for congratulation that under the auspices of Georgina Countess of Dudley a complete system of aid for those who require it has been quietly organised in such a manner that without publicity and without delay, each nurse will find herself the object of individual care from the moment of landing in England. She will be able, moreover, to take her rest in her own way. The choice will be given her of seaside or country lodgings, and she may have the additional comfort of inviting a relative to accompany her, the whole expense being defrayed by Lady Dudley's Fund for a period of six weeks or two months, as required. Lodgings have been selected after personal inspection in the leading watering-places in the United Kingdom, and special provision has been made for those who may have been dispatched from the colonies and have no friends here. This is far better than private hospitality, which, though readily forthcoming, could not obviously have afforded the same facilities for absolute rest.

### "THE DUTCHMAN DIDN'T MIND."

Writing to us from Gwelo, Rhodesia, a nurse says: "Do you care to hear how we, in this dull little town in far away Rhodesia, celebrated the relief of Mafeking? We heard the good news early in the day, and very soon flags (made, alas! in anticipation weeks ago) were flying from nearly every little tin shanty; business—the little there is—was suspended, and Gwelo, as far as empty pockets and hard times will permit, was *en fête*. In hospital we determined to celebrate the occasion by a real good "scoff," and this matron was able to do, thanks to the kindness of an old patient, who sent guinea fowls; and a local gentleman, who presented champagne. Old Antonio, the coolie cook, rose nobly to the occasion, and at seven p.m. the doors between the three small wards were thrown open, the convalescents being seated at the table, and the wards looking quite smart with the bright red blankets and the picturesque garb of some of the convalescents. Doctor, matron, nurses, and orderly being assembled, the Kaffir boys crowding by the windows wondering at this latest freak of 'Missis.' Our Doctor, in a few concise, well-chosen words, proposed 'Our Queen and Empire,' which was enthusiastically drunk. Next came 'Our Troops—English and Colonial'; and last, though not least, with three cheers, and yet another, to the tune of 'He's a Jolly Good Fellow,' we toasted 'Baden-Powell, the greatest hero of them all in South Africa.' We had one Dutchman in the ward, but he drank his champagne philosophically, and didn't seem to mind. We had also eight Australians on their way to the front; they, poor fellows, are very sick at being too late for Mafeking, but hope to be in the front at Pretoria."

### PENSIONS FOR THE NURSES AT ADDENBROOKE'S.

A very wise decision has been arrived at by the Governors of Addenbrooke's Hospital, Cambridge. The weekly Board having made some recommendations for the formation of a pension scheme, they have been referred, at the instance of Dr. MacAlister, to the Advisory Committee for consideration. Dr. MacAlister and his supporters had not a word to urge against the principle of pensions, but they suggested the propriety of allowing the body responsible for the finances of the hospital to deal with the matter, and the former said that it might be best to encourage their nurses to join the Royal National Pension Fund for Nurses. This might easily be done on the same lines as at Guy's and other hospitals, and the Advisory Committee at Addenbrooke's would find it worth while to ascertain the results in the cases of institutions affiliated to the Pension Fund. Pensions for the nurses of every hospital are most desirable, but a brand-new and independent scheme for each is not required when there is a central organisation which, as it has been proved so often, offers every possible advantage.

### THE NEW NURSES' HOME AT BRISTOL.

The nurses at the Bristol Royal Infirmary must be congratulated upon their new home, which affords provision for practically the entire staff. There is bedroom accommodation for 93 nurses, and to a great extent the nurses have separate rooms. There are also recreation and reading rooms, superintendent's sittingroom, cloak-room, box-room, and numerous bathrooms, in addition to kitchen and domestic offices. The bedrooms of the new part are arranged four storeys high on each side of an L-shaped corridor, and they are approached by fireproof staircases. An external fire escape is also provided, and the roof and corridor are of fireproof construction. The heating of the building is by hot-water pipes, and the ventilation of each room is effected by flues communicating with a central extracting shaft. The whole of the building is lighted by electricity, and the cost, including furnishing and repairs, has been about £8,600. By the acquisition of an adjoining piece of land it has been found possible to form a spacious garden, sufficiently large to provide a tennis court and other means of outdoor recreation.

### NURSING THE SICK AT SEA.

At the annual conference of the Matrons' Council last week, the Comte de Cardi urged the importance of nursing the sick at sea in a more adequate fashion, and advocated legislation compelling all passenger vessels to provide hospital accommodation, and a trained nurse as well as a doctor. In France, he pointed out, such a law has been in operation for thirteen years. That the sick at sea require proper nursing is obvious, and that passenger ships should have on board a trained nurse is highly desirable. Whether compulsion is wise is fairly arguable. It would be better if the great shipping companies with one consent led the way voluntarily and made the provision in their own interest.

### A RECENT APPOINTMENT BY THE BIRKENHEAD GUARDIANS.

We have received several letters expressing astonishment at the appointment of a superintendent nurse by the Guardians of the Birkenhead Union Infirmary, and asking whether it can be justified. The General Order of the Local Government Board in reference to the Nursing of the Sick in Workhouses, dated August 6th, 1897, leaves no room for doubt as to the duty of the Guardians. According to clause 3, article 3, "Any superintendent nurse appointed after the commencement of this Order shall, unless we dispense with the requirement, be a person qualified for the appointment by having undergone, for three years at least, a course of instruction in the medical and surgical wards of any hospital or infirmary being a training school for nurses, and maintaining a resident physician or house surgeon." If these conditions have been observed, the Birkenhead Guardians must be held blameless; but if not, it will become the duty of the Local Government Board to institute inquiries.

### THE NEEDS OF THE LONDON SCHOOL NURSES' SOCIETY.

The report of the annual meeting of the London School Nurses' Society, which appears in another column, shows the kind of work it is doing. But we regret to see that it has not, so far, met with the support it merits, and that the treasurer declares "it will be bankrupt by Christmas unless help is forthcoming." It is true that he added a saving clause, and hinted that the continuance of the entertainments given might avert such a catastrophe. But the solvency of the society ought not to depend upon entertainments. We hope that its claims to systematic financial support will be recognised, and that the assured income of £500 a year, for which the Committee ask in order to keep eight nurses regularly employed, will soon be forthcoming.

### MATERNITY NURSING.

The rush of women to train as maternity nurses, in both the monthly and midwifery branches, increases. We are continually receiving inquiries as to the cheapest and best courses of instruction, and the pressure upon the lying-in hospitals is very great. At York Road, for instance, the vacancies for students of monthly nursing are booked for six months in advance; whilst those for midwifery pupils are filled 12 months beforehand. Only chance vacancies, caused by the failure of an accepted candidate to attend her appointed course of instruction, are available for seekers after immediate training. The least expensive way to acquire a proper training is provided by the Midwives' Institute, in the shape of lectures for the candidates, and arrangements for them to accompany a district nurse and be instructed by her in practical work. The best mode is, however, to devote three months entirely to the work, and to study at one of the maternity hospitals. When it is remembered that the fees charged by these establishments include board and residence, it will be realised that there is little or no profit to be derived by the training institution. All maternity nurses are eligible as members of the Midwives' Institute and of the Royal National Pension Fund for Nurses.

### SHORT ITEMS.

The anniversary festival of the Meath Home of Comfort, Godalming, will be held on Thursday, July 26th. After service in the parish church there will be a gathering of associates and friends at the home, and the industries of the patients will be exhibited and offered for sale.—The Nursing Aid Society, which we mentioned a year or two ago as doing useful work in diffusing a knowledge of useful dieting in the homes of the poor, has developed by degrees into the Bristol Cottage Hospital, with eight nurses, who work free among the very poor, and at moderate fees to others. The large amount of maternity work, and the very fair number of patients—mostly women and children—at the out-patient departments, gives scope for the training of nurses for private or district work.—By command of her Royal Highness the President of the Royal British Nurses' Association, a meeting of the General Council will be held at 11, Chandos Street, Cavendish Square, on Friday, July 13th, at five p.m. All members are invited to attend, but only those who are members of the General Council are at liberty to speak or vote.—On Monday the Princess of Wales paid a private visit of inspection to her hospital ship on its arrival at Southampton with invalids from South Africa. On completing her tour of the vessel, and after speaking a few words to each of the 170 patients, her Royal Highness expressed her entire satisfaction with the arrangements.—Nurse Mill, who has just severed her connection with the Govan district of the Victoria Jubilee Nursing Association, was the first Jubilee nurse to be stationed in the burgh. During her five years of service she won the esteem of a large circle of friends and patients.—The foundation-stone of a Nurses' Home for Denton, which is to cost about £700, and is to be the gift of Mr. William Lees, has been laid by the wife of the donor. After the ceremony there was a huge cycle parade and trades demonstration in aid of the Denton Nursing Association.

### Lectures on Medicine to Nurses.

By H. A. LATIMER, M.D. (Dunelm), M.R.C.S. Eng., L.S.A.Lond.; Consulting Surgeon, Swansea Hospital; Past President of the South Wales and Monmouthshire Branch of Brit. Med. Assoc.; Past President of the Swansea Medical Society; Hon. Life Member of the St. John Ambulance Association, &c.

### THE PUBLIC NURSING SERVICES.

THERE are, in my opinion, several directions in which I would like to see the nurse employed more fully than she has been in the past. The gaps are being filled up slowly, it is true, but it is surely time that they should no longer exist at all. I refer to a good nursing service in the workhouse infirmaries throughout the country, and to the appointment of district nurses in towns and villages all over the land. In the case of the union infirmaries it has been too long a custom to supply a somewhat inferior medical and nursing attendance. Such a policy on the part of the responsible authorities is an exceedingly short-sighted one, for it is surely important for the welfare of the community that the poor folk who find their way there should, if their ailments are of a curable nature, be restored to health at the earliest opportunity, so that they may again betake themselves to the wage-earning classes. If the remuneration of the union medical officer were raised to a satisfactory amount good men would as eagerly compete for these posts as they do for other paid ones, and we should have workhouse medical staffs composed of men of the highest attainments, with a corresponding advantage to the poor people who would be their patients. But the scale of pay now doled out to the Poor Law medical service is altogether inadequate to obtain men of this sort, and, except in some favoured places where the authorities are rising to the needs of modern requirements, the emoluments for such services are not based on a satisfactory scale. If more, and better, medical men were appointed, at better salaries than at present given, higher work would be done than is the case at present; and as a part of this higher-class work the nursing service would be improved from its present state.

The introduction of the district nurse has been a great boon and blessing to the community at large. I can personally testify to the help she has been to the medical man in his attendance upon the poor. Figure to yourselves a patient lying ill in wretched surroundings, suffering from some grave ailment such as acute rheumatism, enteric fever, or advanced phthisis, or—and here the condition is if anything more serious still—with some surgical affection, accompanied by a suppurative process or necessitating the carrying out of skilful surgical dressing. His medical attendant cannot well be long with him, for he has a large number of patients to see in the day in scattered places, it being quite a fact that the poorer the patients he attends the larger number he will have to visit, and the smaller the remuneration will be for his work. He goes in, then, to see his patient, gives his directions about food, medicines, and nursing, and then resumes his round. But what if the invalid be lying with unchanged bed-linen, with unwashed body, and with so bad or careless an attendance upon him that he is sure to suffer from complications of his illness which are avoidable under proper precautions. In this regard I may say that some of the most formidable bed-sores I have ever seen have occurred in cases of enteric fever in badly nursed patients at their homes in poor surroundings.

Or again, let me quote a type of case where immense good results from the attendance of a skilled nurse. Only recently I had occasion to open an abdominal abscess, the result of a peri-typhlitis. A large quantity of highly offensive pus was evacuated by my knife, and I made the first dressing with all attention to antiseptic details. But drainage had to continue, and dressings had to be removed and replaced often, whilst the toilet of the patient had to be attended to. Thanks to the work performed by a skilled district nurse, this was done regularly and satisfactorily. I leave you to imagine what would have taken place if the patient had been dependent upon friends for this work of skill.

The rich can afford to have nurses in their houses, to feed and to lodge them, and to pay the necessary fees for their attendance; the poor can often now be attended satisfactorily in their own homes by the help of district nurses, where formerly they would have been compelled to have been removed to hospitals, simply because they could not have been satisfactorily nursed where they lay. Looking all these facts in the face, we are justified in regarding the trained district nurse, who is a product of modern ideas, as one who performs a highly valuable function in the national life.

It is somewhat curious to notice how long a time elapses before a public improvement of one sort or another is adopted after its necessity has been plainly shown to have existed. This one of the supply of skilled nurses to the poor at their own homes is one in point. It must have been apparent to all charitably-minded people who have done any visiting amongst the poor that it would be a work of the highest importance to the latter if they could be supplied with some help of this sort when the necessity for it arose, and yet it is only of comparatively recent date that such aid has been forthcoming. The comfort—let alone the direct advantage from a health-giving point of view—of a skilful nurse appearing on the scene in a small home where the daily toils of life give no time for extra work resulting from illness in a house is immense, and I feel convinced that the scale of ill-health is often turned in favour of recovery in such cases by proper nursing, where otherwise it would have gone in the other direction. So I have come to regard the Queen Victoria's Jubilee Institution of Nurses as one of the most beneficent memorials of the reign of this great Sovereign which could possibly have been made. We are now living in an age where all knowledge is becoming systemised and specialised, and where there is a loud and increasingly powerful cry for technical education in the various callings of everyday life. Nowhere is this seen more forcibly than in the career of a nurse. Her training now has to be full and exhaustive, and when she has successfully emerged from her stage of pupilship she has many posts open to her in which the public is unwilling that she shall be intruded upon by unskilful competitors. The most recent and most marked instance of this fact is what has just been occurring in South Africa during the war which is at present being waged there. In the fervour of patriotism, and, as I fear must often be the case, under the desire for some new excitement, a rush of well-placed ladies to Natal and Cape Colony occurred, and the work of the surgeons and professional nurses at the military hospitals there was seriously interfered with by these inexperienced and over-zealous persons. The time has gone by when—as in the days of the Crimean War—our Army Medical Service is in need of amateur helpers of this sort. Now the army, colonial, and Indian medical administrations are organised in the matter of attention to nursing aid, as well as in the provision of physicians and surgeons, and no place can be found for unskilled helpers, who are worse than useless in such situations as the one we are considering. The letters and speeches from men who have been doing practical work at the seat of war of late have shown very conclusively that only those nurses who have been properly trained can be made use of there; and what is true of work in that part of the world is true of systematic work in civil life. My concluding remarks on this subject, then, are these: Let the authorities in charge of the education of nurses see that a high standard of attainments is exacted from those who adopt this calling, and let those who take up the same work with the feeling that by qualifying themselves well for their duties they will be doing what cannot fail to secure for themselves the fullest recognition by the public at large of their utility and value.

### Functions of the Week.

### THE QUEEN'S NURSES.

### Presentation of Badges by Princess Henry of Battenberg.

At about half-past two on Thursday afternoon last week people in Victoria Street noticed groups of nurses in dark blue uniforms going towards the Scottish Drill Hall in Buckingham Gate. They were the Queen's nurses ("And how kind it is of the Queen to spare them to us!" as a poor woman once said), and they were about to receive their badges from H.R.H. Princess Henry of Battenberg. Places had been reserved for them, and they made a bright and pretty picture; the superintendents in navy blue, and the nurses in blue cotton dresses and white aprons. The other side of the hall was well filled by interested spectators.

On the stroke of three the Princess arrived, attended by Major the Hon. F. L. Colborne and Miss Bulteel; she was conducted to the platform by the president, the Rev. A. L. B. Peile. A beautiful bouquet of red roses and lilies of the valley, tied with a broad red ribbon, was presented to Her Royal Highness by Miss Susan Cottrell, whose name stood at the head of the list of newly-appointed Queen's nurses. There was not a great deal of speechifying, and the whole ceremony occupied only forty minutes.

### The Growth of the Movement.

The President said that people had begun to see what a valuable institution the Queen had set on foot. It had grown marvellously, and this was due largely to the interest taken in it all along by Her Majesty. With regard to the nurses themselves, he believed that no nurses worked with greater zeal and earnestness than the Queen's Nurses. There may have been prejudices against them sometimes, but they were breaking these down, and leaving good influences behind them wherever they went. Mr. Peile alluded to the likelihood of three nurses going shortly to an important colliery town, and said that half the amount required for their support was to be contributed by the colliers themselves, who gave gladly, realising the enormous benefit of having skilled women among them.

### Funds Wanted.

Of course more funds were asked for by the hon. treasurer; it would be unusual, to say the least, not to ask; and in a brief speech Mr. Boulton acknowledged the kindness of the Music Hall authorities in having given entertainments for the benefit of the institution. Their contributions had amounted to at least £400. The institute was spending from £9,000 to £10,000 yearly. He also thanked the district associations for their loyal support, which might be likened to that of the colonies towards the mother country.

### The Nurses.

Addressing the nurses assembled, the Hon. Treasurer then said: "It is customary on these occasions for someone who is more or less an outsider, or at any rate not a medical expert, to say a few words to you. Nobody knows less of nursing than I do; but I do know something of what the Queen's Nurses do for the poor; how they bring sympathy and light into poor people's lives. Nurses, the Queen's daughter is going to give you the Queen's badge; it comes straight from the Queen, and it is given you because you have the valour to go forward and fight against ignorance and disease; to receive it is as great an honour as to receive the Victoria Cross. You are taking it upon you at a time when the reputation of the Queen's Nurses is very high; and we want you to wear also in your hearts kindness and sympathy for those amongst whom you work. We shall always praise you very highly and pat you on the back; but be sure that your Queen and country will exact from you the utmost loyalty and diligence and devotion."

### The Function.

Then the badges were given by the Princess. The nurses came up to the platform in order as their names were read out, received their badges, made their curtseys, and went back to their seats.

First, six silver badges were given. These were for the new superintendents of district nursing homes, as follows: Kate Emma Barlow, Leeds; Lizzie Odell Carter, Reading; Annie S. Dunne, Hull; Jessie H. Matthew, Camberwell, London; Rebecca R. Piercy, Huddersfield; Alice Wall, Liverpool (Overton Street Home). Next, 48 nurses received bronze badges and brassards on their appointment as Queen's nurses; 10 certificates were given on the completion of two years' satisfactory service; and 4 superintendents and nurses received "leaving" badges and certificates (these badges are for not less than six years' satisfactory service as Queen's nurses.

The other speakers were Sir Dyce Duckworth, Sir Fleetwood Edwards, and Sir Arthur Arnold; and the company included a large number of the council of the institute.

---

### GUY'S HOSPITAL GARDEN PARTY.

Wednesday last week was Prize Day at Guy's Hospital. The invitations of the governors and medical staff to the ceremony were widely accepted, and the picturesque buildings and grounds of the famous old hospital were thronged with visitors throughout the afternoon. The distribution of prizes by the Earl and Countess of Pembroke took place in the theatre in the new school buildings, Mr. Cosmo Bonsor, M.P. (treasurer), presiding. In congratulating the successful students, Lord Pembroke took occasion to note the fact that nearly one hundred medical men and nurses trained at Guy's had gone to serve their country in South Africa, and the hospital had to regret the loss of two of their surgeons on the battlefield, while six had died from enteric, and others had suffered from wounds and sickness. Afterwards the visitors found their way into the wards, looking bright and festive with charmingly-arranged masses of flowers, and over the new laboratories, museums, and college. The newly-fitted surgical wards contained much calling for admiration, with their terrazzo floors, tiled tables and lockers, glass and metal dressing tables, &c., the whole carried out with a regard for artistic effect in the matter of colouring, and a sanitary fitness from the aseptic point of view that was eminently satisfactory. The band of the Scots Guards played in the park, and refreshments were served in the quadrangles of the surgical buildings. Altogether the function was extremely pleasant, and afforded an excellent opportunity for the hospital-subscribing public to note that Guy's is keeping well to the fore in the matter of structural improvements. It is satisfactory to see that the walls of the new and much-needed nurses' home are slowly rising in their appointed place.

---

### THE ST. PANCRAS INFIRMARY.

Following their annual custom, the Guardians of St. Pancras issued invitations for a general inspection of the infirmary on Dartmouth Park Hill on Friday, their principle being the very right and wise one that the ratepayers who support these institutions should be given an opportunity of seeing for themselves how the sick and infirm of the parish are cared for and tended. There is nothing very new to note with regard to the St. Pancras Infirmary, one of the older of the State hospitals, built on the more unattractive lines of past years, and in no way to be compared to the more elaborate and better planned of quite modern infirmaries. The home for the nursing staff is the most recent addition to the building, and contains an especially comfortable and pleasant sitting-room, and a convenient dining-room. It seems a pity that the essential necessity of a separate bedroom for every nurse was not generally recognised when the home was planned, the probationers having to share two and three-bedded rooms. In other respects every adequate provision is made for the comfort of the nurses. A good plan has been followed at St. Pancras in the classification of cases, one or more wards having been set apart for consumptive patients, with a small ward which can be used for the worst cases if necessary. The nursing at St. Pancras is in the able hands of Miss Moir, one of those matrons to whom the Poor Law service owes much for patient and untiring work. The nurse-like appearance of her staff invariably calls for admiration. The children's ward was a chief centre of attraction on Friday, and here was to be admired a new overmantle—a much-appreciated decoration—painted by a kind friend of the infirmary.

### Presentations.

ADDENBROOKE'S HOSPITAL, CAMBRIDGE.— Mrs. and Miss Newman have been presented on the occasion of their resignation of posts of sisters at Addenbrooke's Hospital with cheques for a substantial amount. The presentation was made by Sir R. P. Fitzgerald, M.P., and several governors spoke in appreciation of the valuable services rendered by the two ladies during a quarter of a century. The subscriptions came more from patients, for in the county the name of Newman is almost a household word, and the Governors have granted the ladies the utmost amount they have ever granted to nurses on retiring. In addition to the presentations for which subscriptions were invited, cheques were given by the past and present nurses.

CHRISTCHURCH NURSING ASSOCIATION.—Last week an interesting presentation took place in the Village Room, Stanpit. A silver sugar basin and tongs, and a silver cream jug and butter knife were presented to Nurse Brough by the Rev. H. J. Pearson, M.A., in the name of upwards of fifty of the residents in Purewell, Burton Road, Stanpit and Mudeford, who wished to testify their affectionate appreciation of the many kindnesses and skilful care they had received from her. The money was contributed exclusively by poor people whom Miss Brough had nursed.

POPLAR AND STEPNEY SICK ASYLUM.—Dr. E. M. Goldie, who has just resigned his appointment at this institution, was last week the recipient of a massive solid silver flower bowl on an ebony stand, by the nursing staff, male officers, and servants.—A presentation was also made to Dr. R. Ross Sutter, M.D., of a massive brass inkstand from the sisters on the female side of the hospital on the occasion of the doctor leaving their wards to take up duty on the male side.

MARYHILL NURSING ASSOCIATION.—Last week a solid silver tea service enclosed in a handsome oak box was presented to Nurse McNaughton upon the occasion of her leaving Maryhill for a position in the North of England after three years' district work.

### Appointments.

WATTON VICTORIA HOSPITAL.—Miss Amelie J. Allen has been appointed Matron. She was trained at the Royal Infirmary, Derby, and Union Infirmary, Derby, and has since been Queen's District Nurse, Banbury.

### The Princess of Wales's and other Nurses for South Africa.

ON Saturday twenty London Hospital nurses, selected by the Hon. Sydney Holland at the instance of the Princess of Wales, will sail for South-Africa. Their names are as follows: Miss H. O. Luckie, sister in charge, and the Misses M. S. Baines, E. Baldrey, L. Bristow, E. Fry, A. Gore, C. Hanbury, M. M. Holloway, L. Humphreys, I. Lawson, B. E. Lloyd, C. E. E. Marsh, K. Parminter, J. B. Robertson, E. Smyth, V. P. Squire, M. E. Tate, C. E. A. Thorpe, A. Thomas, and E. Whistler.

MISS HATTIE OLESON LUCKIE, who is better known to her hospital friends as "Sister Gloucester," has been a member of the "London" nursing staff, serving in various capacities since May, 1890. She enjoys the entire confidence of the matron.

MISS THORPE and MISS TATE are old "Londoners" now working at the Metropolitan Hospital.

MISS PARMINTER, MISS THOMAS, and MISS ETHEL FRY are also old "Londoners" now filling appointments elsewhere.

MISS CECIL HANBURY left the "London" comparatively recently, and has not taken up any fresh nursing duties until now.

The remainder are all sisters and nurses who are at present valued members of the nursing staff at the London Hospital.

### THE IMPERIAL YEOMANRY HOSPITAL.

On Saturday the following twenty-nine nurses sailed in the "Dunvegan Castle" for service in the Yeomanry Branch Hospital in addition to those who left a week earlier: Miss F. H. Barry, Miss E. M. Buchanan, Mrs. T. A. Cobbold, Miss E. A. Cowley, Miss H. A. Cruickshank, Miss A. J. Douglas, Miss F. K. Fitzmaurice, Miss E. H. Headley, Miss F. M. Hoggins, of St. John's House, Norfolk Street, Strand; Miss M. E. Ireland, Miss E. Johnson, Miss E. M. Kent, Miss M. Mavius, Miss Meade, Miss M. S. Milne, Royal Infirmary, Dundee; Miss G. C. Moxon, Royal Portsmouth Hospital; Miss M. McLeish, Miss MacLeod, Miss H. M. O'Connor, Miss E. O'Neill, Miss B. Shepley, Miss A. S. Siddons, Miss C. Strahan, Miss E. Timbrell, Miss A. E. Turner, Miss K. Webb, Western Hospital, Fulham, S.W.; Miss D. West, and Miss H. M. Young.

MISS EDITH A. COWLEY was trained at St. Bartholomew's Hospital for three years, afterwards serving as staff nurse. She has since been sister at Salop Infirmary, Shrewsbury, and sister at the Royal Infirmary, Derby.

MISS DINAH WEST was trained at Crumpsall Infirmary, Manchester. She has since been charge nurse at Kildare Infirmary, temporary assistant nurse North Dublin Union Infirmary, and, from November, 1889, superintendent nurse of the Protestant Infirmary, North Dublin Union.

MISS HELENA MARY YOUNG was trained at the Hospital for Women in Soho, and the Metropolitan Hospital, where she has also been staff nurse. Since March, 1898, she has been attached to St. John's House, Norfolk Street. She holds Mrs. Creighton Hale's massage certificate.

All the Yeomanry nurses are paid at the rate of £40 per year, with £20 bonus on returning home.

### To Nurses.

WE invite contributions from any of our readers, and shall be glad to pay for "Notes on News from the Nursing World," or for articles describing nursing experiences, or dealing with any nursing question from an original point of view. The minimum payment for contributions is 5s., but we welcome interesting contributions of a column, or a page, in length. It may be added that notices of entertainments, presentations, and deaths are not paid for, but, of course, we are always glad to receive them. All rejected manuscripts are returned in due course, and all payments for manuscripts used are made as early as possible at the beginning of each quarter.

### Annual Conference of the Matrons' Council.

THE third annual conference of the Matrons' Council of Great Britain and Ireland was held on July 5th and 6th in the Medical Societies' Rooms, 11, Chandos Street. Miss ISLA STEWART, matron of St. Bartholomew's Hospital, and President of the Council, was in the chair. The following ladies read papers on the first day: Miss Julian, Mrs. Lancelot Andrews, and Mrs. Bedford Fenwick.

The PRESIDENT opened the conference. In a short speech she reviewed the work of the association during the past year. The number of members had risen to 104, with nine honorary members. Amongst the latter were some American and Colonial matrons. Three important pieces of work had been carried out by the Council during the past year: (1) An International Council of Nurses had been founded, with Mrs. Bedford Fenwick as president; (2) a resolution had been sent to the University of Durham, suggesting the foundation of a chair of clinical nursing; (3) resolutions had been sent to the Secretary of State for War and the First Lord of the Admiralty, suggesting that a trained nurse should be attached to each of those Government offices which undertook any nursing work.

### THE POOR LAW NURSING SERVICE.

Miss E. JULIAN, matron of Croydon Infirmary, read the first paper upon "The Poor Law Nursing Service." Pointing out the great facilities for training in infirmaries, she showed the difficulties against which the infirmary nurses have to contend. They lack the status of hospital nurses, proper medical instruction, and a regular curriculum. In small infirmaries the nursing superintendent is under the control of the master and matron, in large ones she is completely subordinate to the medical superintendent, and is also often under a ladies' committee. It frequently depended upon the medical superintendent whether her position was that of a toad under a harrow or of a self-respecting individual. In the discussion that followed Dr. TOOGOOD, Mrs. FINLAY, Miss McCLURE, and Mrs. BEDFORD FENWICK took part. General opinion urged central examinations by a Board and the granting of recognised certificates to infirmary nurses.

Mrs. ANDREWS, late superintendent of the Gordon House Home Hospital, read a paper on "The Necessity for a Nursing Department in all Government Offices dealing with the Nursing of the Sick." She urged that the nursing department should be under the control of a trained nurse who had been a matron, this lady to be associated with the Director-General, and to act as administrative nursing officer. Mrs. Andrews further insisted that the Army Nursing Reserve requires a trained and experienced head. The PRESIDENT, Mrs. BEDFORD FENWICK, and others took part in the discussion that followed.

### THE CHANGES OF THE CENTURY.

Mrs. BEDFORD FENWICK, in a paper on "The Celebration by Trained Nurses of the New Century," began by reviewing the changes that have taken place in this century with regard to women's work. She spoke of the newly inaugurated International Council of Nurses, founded to "further the efficient care of the sick and to secure the honour and interests of the nursing profession." "The Council," she said, "sought to provide means of international communication and hospitality between nurses, and to provide opportunities for nurses to meet from all parts of the world." Mrs. Fenwick then gave details of a proposed twentieth-century celebration to be held in Buffalo, U.S.A., next year, to which invitations to the nurses of all nations are to be sent. The following resolution closed the paper: "That the Matrons' Council take steps to form a sub-committee to facilitate the attendance of nurses at the twentieth century celebrations." In the discussion that followed the financial difficulties of the journey, &c., were pointed out.

### STATE REGISTRATION OF NURSES.

On Friday three papers were read on the subject of "State Registration for Nurses." The first, from the doctor's point of view, was by Dr. TOOGOOD, medical superintendent of the Lewisham Infirmary. He considered registration necessary for the good of the nurse and the protection of the doctor and the patient. Many untrained nurses were foisted on the community—hence one necessity for registration. This would have to be carefully carried out, lest there should be any risk of dual control in the sick-room, and the nurse usurp too much authority. There was often already too much tendency on the part of nurses to minimise the importance of the doctor. If State registration can be brought about, Dr. Toogood thinks that no untrained nurse should be admitted, but only those who have at least the minimum of training, though nursing need not be restricted to these. He finally begged nurses to close their ranks and reconcile factions, saying that registration would not come if the present divisions continue; and he advised that members of Parliament should, through their constituencies, be "worried" about the question of State registration.

The PRESIDENT then read a paper by Miss Poole, matron of Blackburn Infirmary, upon State registration from the nurses' point of view. Miss Poole thinks that nurses now occupy an anomalous position, whilst registration would give them a recognition as a profession. "Doctors," she observed, "are legally qualified and registered. Why not nurses also Women give their money and their powers to learn to nurse the sick; then they should be recognised and registered. Private homes often take untrained nurses, the Army Nursing Reserve has not been free from blame. Registration would remedy these evils. Public opinion should be educated to see the advantage of it."

Mrs. EMILY CRAWFORD's paper gave the views of the patient upon the same subject. She considered nursing the greatest of all professions, and one made by women. She thought that nurses had been maligned and misrepresented. She advised them to ask for what they wanted until they got it.

The PRESIDENT and Mrs. BEDFORD FENWICK both urged the advisability of State registration as a protection to the profession, to the public, and to the medical man. A resolution to the effect that State registration of nurses is a matter of national importance was put to the meeting and carried unanimously.

### NURSING THE SICK AT SEA.

The Comte de CARDI, speaking from a long knowledge of tropical countries, suggested in his paper the great importance of "Nursing the Sick at Sea" in a more adequate fashion. Invalids and others returning from tropical climates frequently required nursing on board ship. The Comte considered that all passenger vessels should be fitted with a hospital, and should have a doctor and a trained nurse on board. The French had passed a law to this effect in 1887 for their vessels. Could not England do what France has done? This was again a matter on which to rouse public opinion. In conclusion, the Comte paid a warm tribute to Miss Mary Kingsley, adding that great interest had been taken by her in the question of nursing the sick at sea, and expressing a hope that some of the money to be raised for a memorial to her might be used in this direction. The paper was strongly supported.

### London School Nurses' Society.

ANNUAL MEETING.

The second annual meeting of the London School Nurses' Society was held in the Board Room of the School Board Offices, Victoria Embankment, on Friday. The chair was taken by the President of the Society, the Countess of Aberdeen, who in her brief opening remarks spoke of the importance of arresting the beginnings of disease, and alluded to the good work being done by the society. A letter was read by her from Mr. Treves, regretting that on account of his numerous engagements he was unable to be present.

The annual report and balance-sheet were taken as read, and their adoption was moved by the Bishop of London.

The Scientific Basis of the Work.

The association, Dr. Creighton said, was small and young, but these were not objections to it. It was an association whose work could be brought into definite compass with very little effort, and it was rarely that any society was able to place its aims so definitely and within such small compass before its members. What, he asked, is the capital of any country? Certainly the physical health of the people. Cleanliness was next to godliness; some people thought that godliness did not make so much headway as it should, because it was difficult of achievement. But it was within everyone's reach to be clean. One would have thought, the speaker went on, that when once a person had had a bath, or even washed his hands, he would be inspired by a wild desire to take a bath perpetually, or at least to be perpetually washing his hands. But it was not so, and he had often pondered on this line of consideration, which he found very fruitful of results in meditating upon human nature. "I have frequently," he continued, "examined boys of what I suppose I must call our own class, and have asked them at what period they begin to wash their hands for pleasure, and I have found that it does not occur until sixteen as a rule. And if this is so, what must be the case with those who have had fewer advantages? It is an education which must be provided. The mere fact of undergoing a periodical inspection, and of hearing remarks made on one's personal appearance, brings into life a new standard—provides new things to think about. We all know the maladies that prevail among dirty surroundings, and how infectious diseases are no respecters of persons. They are the results largely of bringing children together in great numbers; the work, therefore, of the association has a scientific basis, and is of great educational importance. There was one remark in the report which he criticised. The committee appealed for an assured income of £500 a year with which to keep school nurses at work, on the ground that the money so spent would be returned a thousandfold in the emptying of the fever hospitals and infirmaries, not only by decreasing the spread of infection now, but in practically inculcating cleanliness on the children in such a way that in the future dirt and disease will be alike foreign and abhorrent to them. He never liked to give any definite promise that any definite work undertaken would bring about any definite results; he preferred to put forward the claims of the society on its own merits.

The Value of the Work.

Dr. Brudenell Carter seconded, and gave it as his opinion that the proverb, "Cleanliness is next to godliness" really meant "It is not within the power of everyone to be goodly (*i.e.*, good-looking or beautiful), but it is in everyone's power to be clean." The work of the association was, he said, of great educational value. What the mother sees and the child feels every day is an object lesson. The child knows that if its burn or its cut finger is kept clean it gets well. People had been heard to make the criticism that the work of the nurses trespassed on that of medical men; this was quite a mistake; the nurse's work was quite distinct from a doctor's. He had seen some thousands of the children, when conducting investigations as to eyesight some few years ago, and he could, therefore, speak with certainty of the value of the work being done in the schools.

The Staff of the Society.

Sir Charles Elliott moved the re-election of the officers. He had never heard of the criticism alluded to by Dr. Carter, but he had known of school managers, badly bitten with political economy, who had made the objection that the work of the nurses interfered with the responsibility of parents. It surprised him, but he thought that there was in the objection a certain amount of truth. He had seen the nurses at work, and had felt what a good thing it would have been if the mother of the children could have been there. (This suggestion, a subsequent speaker said, had already been acted upon.) He wished to make one suggestion which arose out of the report, namely, that it was very desirable, there being so few nurses (three during the past year had been at work; the Association asked for eight, and this was a very small number), that the worst schools should be selected for their visits. He noticed that two of the schools had hardly any cases of heads or eyes. Burns and cuts were not evils found exclusively in the worst schools, whereas the diseases caused from dirt were.

The Rev. H. Russell Wakefield supported the motion, and spoke of the valuable work done for the Association by its hon. secretary, Miss Honnor Morten, and the other officers. The work of the society ought to be greatly extended. He knew of at least one school in which it was perfectly impossible to have enough nurses. He had asked the head mistress of the girls' department whether bad eyes were increasing; she had replied, "Yes, undoubtedly, through contact with one another." How great an advantage, then, to have skilled persons to cope with the evil.

The Need of Help.

The Hon. Lyulph Stanley, in moving a vote of thanks to Lady Aberdeen, said that voluntary effort had often to go before municipal, and he hoped that the work of the society would help to set a good example.

Mr. Bridgeman, in seconding, took the opportunity of alluding to Sir Charles Elliott's remark about schools No. 3 and No. 4 in the report, in which there were no cases of eyes and heads; Sir Charles had appeared to think that for this reason the nurses ought not to go to those schools. He wished to explain that the reason why there were "no heads and no eyes" was that the nurses had been going there for some time. As treasurer, he would remark that people usually thought that because there was a balance in hand or at the bank a society was in a flourishing condition. Since the account was drawn up he had spent £35 and received £3 10s. The society would be bankrupt by Christmas unless help was forthcoming, especially if the entertainments given during the last year or two were not continued. He added, "Nobody seems to know of the society, though when people do know of it they all think it a very good thing."

### Death in Our Ranks.

We regret to hear of the death of Miss Margaret Elizabeth Henniker, who only completed her three years' training at the Victoria Hospital, Chelsea, in June, 1899. She was hoping to enter University College Hospital the following September for adult training, but was taken ill almost immediately after she left Chelsea with tubercular nephritis, from which she suffered greatly until the day of her death last month.

The death of Nurse Florence Castle at St. Mary's Hospital, Paddington, is announced. Miss Castle was the eldest daughter of Major-General F. J. Castle, a Crimean veteran, and was only twenty-seven years of age.

### Nursing in America.

By an English Nurse.

V.

No time was grudged to the operators' room or to the sterilising of dressings. The surgery was beyond reproach, and their cases did splendidly. One man I shall not forget—a brakesman on the railway. He came in with both legs so badly smashed that double amputation was necessary, one leg being amputated close to the hip. The accident had occurred four hours before, but distances are great and hospitals far apart, and though three pints of whisky had been administered meanwhile, he was in such a collapsed state that first we dare not get him on to the operating table, and then we dare not get him off, but he made a speedy recovery. Another, a young Englishman suffering from appendicitis, who, coming in soon after I went on day duty, was handed over to me as a fellow-countryman in distress, did equally well. No antiseptics of any kind were used, simply sterilisation of dressings, water, and person being thoroughly carried out; in one or two instances a salt solution was used for irrigating.

### The Prevalence of Appendicitis.

By the way, appendicitis is *the* disease over there at present. One hears the word everywhere, in cars, in churches, in theatres, or as one walks along the street. One Sunday afternoon in Boston when I was going down to King's Chapel (a real old-fashioned two hundred year old English church buried down in the business quarter of the city), I counted and found that I had heard the word eleven times in the course of two hours.

### The Linen and the Lavatories.

Linen closets were kept in the most immaculate order, not a fold being out of place, and as often as not it was the matron who saw to them. But lavatories were in a condition no matron of a cottage hospital at home would tolerate, and except that they were rubbed with alcohol, no patient had more than face and hands washed, except in the weekly bath, and that was a kind of movable festival, depending upon whether the nurse had time to give it, and whether the patient was willing to take it. Except the upper classes and such as have taken to English ways, the Americans as a nation are not as fond of "tubbing" as we are. Just as the women used to wonder at my walking powers, so they wondered at my daily cold tubbing. It was superfluous they thought, and I always had a lurking idea that they imagined it was coarsening.

### Hours Off.

Each nurse had an hour off a day (such hour being usually decided by the matron at dinner time) and an afternoon a week. The afternoon was all right, but I soon found I could not go on under such conditions. With rooms and wards at anything from 75 deg. to 80 deg., one had to wear the thinnest of summer clothing; and with the thermometer outside hanging just above or below zero, one could not venture over the door without changing right through, dry though the air was. But coming up tired out with hard physical labour, it was impossible to undress, dress, go out, and then repeat the process in the course of an hour, the consequence being that one could only have fresh air once a week, a state of things to which I had never been accustomed.

### A Victim to Influenza.

In addition to my surgical cases, I had also a case of nervous prostration, requiring any amount of feeding up, and to whom I had to give half an hour's massage twice a day. My patient gained at the rate of four pounds a week, I lost at the rate of three, with the result that when influenza made its appearance among us, brought in by an operation case of my own who was to have been operated upon next day—whose temperature, more from habit than anything else, I had taken on admission, finding it 102·8—I fell one of the earliest victims. I had it pretty severely, and as everyone was down, and the matron at day and her sister at night were heroically trying to fill the gap and do everything single-handed, no nurses being obtainable, I felt that as I had intended leaving at the end of the month any way, the sooner I gave them one less to wait on and care for the better. I did not leave after my nine weeks' stay without regret. The country round was very beautiful, and the violets and roses of the sunrises and golds and purples of the sunsets over the white mountains, were something to remember. True after a while the wide silent whiteness began to depress me. It was so white, so silent, the whole world seemed dead and spring nothing but a chimera, one could not imagine bursting bud and leaf ever coming to that ice-bound land, where the very rivers are indistinguishable from the surrounding country, so fast were they bound in winter's chain. I think the sweetest sound I ever heard was one day in driving over a bridge—that of the far-off sound of running water below. It seemed the beginning of a resurrection of which one had dreamed, but had hardly dared to believe in. I had made friends, too, and received much kindness, but I felt that I should be better in Boston, where I could be among those who knew me, and could have such food as I could eat, for New England fare as one got it up there, pure and unadulterated, did not suit me.

### An Opening for Private Nurses.

Had I cared to wait and go in for private nursing, I could have obtained plenty of employment at 3 dols. a week, for there was but one nurse within a radius of fifty miles. But for this I should have been expected to do as the mother would have done in caring for the children, cooking for the husband, and keeping the house straight.

### Leaving for England.

I had been in bed ten days, and trying to get about somehow for two, when I started, and how I got to Boston I shall never be quite sure. The doctor told me it was madness, but I was perverse and would go, so go I did. They made it as easy for me as they could, doing my packing, and seeing me safely bestowed in a parlour car, under the guardianship of the conductor. Kind friends met me at the other end and saw me safely bestowed in my old rooms, Mrs. D. being luckily able to take me in, till my steamer sailed, and I shall not forget the kindness of those kindly Americans during that last fortnight there. Such doctors as I had nursed for called to see what they could do in the way of building me up, their wives brought jellies, and in two instances insisted on carrying me off for a few days to be under their husbands' care, and, as they expressed it, be properly fed and seen after.

I must confess that it was with very real regret that I left the new world to return to the old. I had got to love the clear, sunny, bracing air, the wonderful atmosphere, and the kind-hearted Americans with whom I had come in contact. The voyage home was much less eventful than the one out, when we had a gale, icebergs, and a fog, by way of breaking up the monotony. My fellow passengers were mostly holiday-makers bent on doing the Exposition, and having a summer in Europe, but I am afraid they must have thought me a most unsociable person, as most of my time was spent in a quiet corner in my deck chair. There was the usual amount of flirtation and friendship. The first few days the onlooker could watch the mutual attractions, the last few, after a spell of rain lasting two days, which left everyone feeling cross and disagreeable, the falling apart process set in, and few landed at Liverpool, I fancy, much better friends than when they left New England's shores.

### Echoes from the Outside World.

AN OPEN LETTER TO A HOSPITAL NURSE.

The news from China at the end of last week was almost as bad as it could possibly be. The legations at Pekin were said to have fallen, and the Europeans who had gone there for shelter to have been killed after in many cases being subjected to hideous torture. The mission stations were reported to have been burnt down, Chinese women as well as men being dragged forth and hewn to pieces, whilst even little children were cut in two by the "Boxers" and thrown into the flames. According to a telegram from St. Petersburg, the members of the Russian Mission had been first tortured by having boiling water poured over their heads, and were then decapitated and cut in pieces. But on Monday we heard more hopeful tidings. For instance, there came a native messenger to Shanghai who stated that on July 2nd the Legations were still standing, and might hold out for some time unless food and ammunition ran short. This was quickly followed by a telegram announcing that a Chinese newspaper had chronicled the arrival of Prince Ching's troops at Pekin, to revictual the Europeans and defend them against the rebels. The untrustworthiness of native reports is evident when the Emperor of China having taken poison, administered by Prince Tuan earlier in the week, and the Empress having gone mad, both reappeared sane and well a little later.

Whilst our hearts were sick with anxiety with regard to the fate of Pekin, the good news about Coomassie did much to help us to look on the bright side of things. The trouble on the Western Coast of Africa, not being on a large scale nor involving even at the worst a great loss of life, had been overlooked by many of us, but those who knew much about the country and the difficulties to be encountered were, I have been told, by no means as sanguine as the War Office respecting the ultimate relief of Coomassie. Now you see that the way the garrison has been saved is not by the arrival of the relief column—which was detained by the heavy rains—but by the brave men taking matters into their own hands and breaking out of their prison. Nothing succeeds like success, but when the small force of 600 fighting men and 700 carriers left the capital the attempt to escape must have seemed to many of them to partake of the nature of a forlorn hope. For some time past rations had been reduced to a minimum, so that the carriers were many of them too weak to carry themselves, much less any serious weight, and the soldiers knew that the number of rebels they might have to face was very large, and that each possessed only 150 rounds of ammunition. A few men had to be left behind to protect Coomassie till fresh help could be brought, and they had to be fed, so out of the slender store Sir Frederic Hodgson, the governor, ordered enough to be left to ration the one hundred men put in charge of the city for twenty-four days, and then, with only sufficient to last the column for two days, the little band turned their backs upon the capital and sallied forth. Happily, the report that the attempt to get out would be made by the Prahsu road, which had our men been more numerous would have been the chosen route, had obtained credence, and the rebels had assembled there in great strength. Still, though the worst was thus avoided, desultory fighting took place all the way to Ekwanta, and no wonder the Governor found it necessary to order two days' halt there for rest and refreshment before he proceeded to the Cape Coast. It is pleasant to read the high praise which he accorded to Major Morris and his two hundred and thirty Hausas, without whose help Coomassie must have fallen, and to remember that just when we most needed them the native troops stood firm and gave us all the assistance possible, though probably there were plenty of temptations to do otherwise. Colonel Willcocks has pledged his word that he will relieve those left behind at Coomassie before July 15th, and even if none of those who escaped with Sir Frederic Hodgson are found sufficiently robust to turn back to help the relieving column, as he asks, Colonel Willcocks will probably prove himself a man of his word.

Although the Duke of Fife is married to the eldest daughter of the Prince of Wales, it is remarkable how both he and his wife manage to avoid the publicity which generally hedges in the Royal circle and enjoy themselves unobtrusively like other less exalted personages. But whenever a gap has to be filled or a kind action has to be done, then the Princess and her husband are to the fore, ready and willing. Unfortunately, the former does not enjoy robust health, and has to be very careful not to over exert herself, so that it sometimes happens that the Duke has to perform a ceremony alone. Such was the case the other day. The bishop of the diocese had promised to lay the foundation-stone of the chancel of a suburban church. He was prevented, and the Duke of Fife consented to supply his place. The way he—quite unintentionally—worried the committee was very funny. He began by arriving before he was expected, and thus escaped the ceremonious reception which would probably have been his share had he not strolled in before due preparations were made. Then he laid the foundation-stone quite "well and truly" I daresay, but not at all according to precedent, for he refused the engraved silver trowel which had been made for him, with the query, "What is it for?" and got along very well without it, rather to the scandal of the well-regulated minds of the officials. But he quite won the heart of one of them by his simple kindness. This gentleman, in seeing to the business part of the performance, somehow managed to graze his wrist. The genial Duke noticed it at once, inquired how he did it and if it hurt much, and added, evidently with the ripe experience of one who had often been present at trifling accidents of the same sort in his little daughters' nursery, "You should put some vaseline on." Evidently the fact that this remedy is not usually kept handy in a place of worship did not strike his Grace.

The other day, in his speech at the third annual meeting of the Central Bureau for the Employment of Women, Mr. Asquith made some very sensible remarks which are worth repeating. His words do not apply with much force to the nursing profession, perhaps, because the fact that training, and good training, is absolutely necessary for success is being so fully recognised on all sides that those nurses who are untrained, or only partially so, are daily becoming fewer. But, I fear, as he says, that the reason why women so often find it difficult to get employment in other prefessions is because, in so many cases, no previous apprenticeship has been served, no effort made to prepare for any particular *rôle* in life, with the result that, as the speaker said, "the common and widespread defect in female work is that there is in it a certain note of the amateur." Until parents recognise the fact that marriage is a career only possible for a few, and that a successful business woman is one who has been well trained, female labour will always be underpaid and employment difficult to obtain even at low remuneration. How little it matters what a woman does so long as she understands her duties was shown by the case mentioned in the hon. secretary's address. A girl, who evidently knew all about horses, applied for a "groom's" situation. Although there were no lady grooms on the books, as she was competent she found the occupation she desired as "coachman" to a lady doctor.

### Everybody's Opinion.

[Correspondence on all subjects is invited, but we cannot in any way be responsible for the opinions expressed by our correspondents. No communication can be entertained if the name and address of the correspondent is not given, as a guarantee of good faith but not necessarily for publication, or unless one side of the paper only is written on.]

### ASSISTANT NURSES UNDER M.A.B.

"An Assistant Nurse" (and one who has two brothers at the front) writes: In reading the account of Mr. Burdett Coutts on fever nursing in South Africa, we who are nurses under the M.A.B. wish to know why they have not been asked to volunteer their services for the front? Is it because they have have not a certificate of three years' general training, although a number of nurses have been fever nursing for years, and many of them would only be too pleased if they had the opportunity to relieve the sufferings of the sick soldiers.

### A SEASONABLE SUGGESTION.

"A Guy's Nurse" writes: Do you think that the various railway railway companies would, if approached on the subject, make a little concession in favour of private nurses, with regard to the amount of luggage allowed when travelling by passenger train? To those of us who in the discharge of our duties are constantly travelling from one place to another, it becomes a serious matter if on every journey we have to pay about 1s. for excess of luggage. We carry nothing with us that is not absolutely necessary, and even then all our little personal belongings, photos, &c., have to be packed away at the institution. Commercial travellers are allowed extra on account of their samples, do you not think that something of the sort might be arranged for us?

### NURSING IN FEVER HOSPITALS.

"E. F." writes: Reading in your columns about some of the troubles caused by the changes of charge nurses, I think it would be nice if the M.A.B. could manage to give their best assistant nurses a certificate for fever nursing, so as to afford them an opportunity to rise by merit to charge nurses for fever work. Many of them stay years under the Board, and are often found thoroughly skilled, experienced women in that particular branch of the work, and yet, because the three years' certificate is lacking, the M.A.B. fail to promote them. They must understand the infectious work better than those who have had only general training, but the latter can take the post of charge nurses at an infectious hospital, knowing nothing about it.

"Nurse C." writes: I read with much interest the article by Dr. Goodall, of the Eastern Hospital, regarding nurses and fever hospitals, and would like, with your permission, to say the following few words. I am a trained and certificated fever nurse, and being much attached to fever nursing have continued that branch of the profession for the past seven years. I applied at the Metropolitan Asylums Board hospitals for the post of charge nurse, but was refused on the ground of my not having a three years' general certificate. Therefore, notwithstanding my being a certified fever nurse, and having had seven years' experience of all forms of fever, their treatment and complications, experience in tracheotomy, &c., I had to make way for those having a three years' general hospital experience, who, perhaps, as I have often found, had never seen a tracheotomy case, used a nasal tube, or douched a throat, as Dr. Goodall states. Other fever trained nurses have had similar experience to myself in this way, and it seems to me that a little reform would not only benefit nurses but fever hospitals and their patients also. Truly a three years' certificate does not make a good fever nurse.

### THE TRAINING OF ROMAN CATHOLICS AS NURSES.

"An Anglican Matron" writes: Can you find room in your valuable paper for a word in reference to a letter in your issue of June 16th on the training of Roman Catholics as nurses? Surely the committee of the "Hospital in Liverpool," which has no vacancies for Roman Catholic probationers, are unaware, first, that probationers are refused on account of their religion; secondly, that when Roman Catholic nurses are admitted they are forced to attend Protestant prayers and services. In a city well known for its liberality in matters of religion and education such narrow-mindedness and intolerance should not exist. It is obviously unfair to disqualify a woman from filling posts for which she is well fitted, simply because she happens to be a Roman Catholic or a Dissenter. My most valued "sister" is a Roman Catholic, and in point of professional skill, kindliness to patients and nurses, tact, conscientiousness, and success in the training of probationers she is a pattern to many who are not Roman Catholics. If such as she are to be debarred from fair play on account of their allegiance to the western branch of the Church Catholic it would be not only a serious loss to the nursing world, but a disgrace to English justice and to our common Christianity.

### Minor Appointments.

Fountain Fever Hospital, Lower Tooting.—Miss Elizabeth Annie Brander has been appointed Charge Nurse. She was trained at the Royal South Hants Infirmary, Southampton, and has since been assistant nurse at the Hospital for Women and Children, Euston Road, London, staff nurse at the Hospital for Women and Children, Birmingham, and assistant matron at the Convalescent Home, Walton-on-Thames.

Camberwell Infirmary.—Miss Clara Stott has been appointed Head Nurse. She was trained at Birkenhead Union Infirmary, and has since been sister in that institution and charge nurse at the Union Infirmary, Richmond, Surrey.

Ingham Infirmary, South Shields.—Miss S. L. Thirlwell has been appointed Sister. She was trained at the General Hospital, Tunbridge Wells, and has since been sister of the children's ward since July, 1899.

Downpatrick County Infirmary.—Miss Lillie S. Fitzgerald has been appointed Charge Nurse. She was trained at the Royal Victoria Hospital, Belfast, and has been staff nurse in the same institution.

Sittingbourne and Milton Joint Hospital.—Miss Sarah J. Lewis has been appointed Charge Nurse. She was trained at Monsall Hospital, Manchester, and has since been staff nurse at Allt-yr-yn Hospital, Newport, Monmouthshire.

### TRAVEL NOTES AND QUERIES.

Switzerland (Pauper).—Yes, there are yet still such places, though fast disappearing, and those terms will not be accepted generally in the season. Try Vevey, on the Lake of Geneva, Hotel de Famille, pension from 4 francs. Also Ballaigues, between Lausanne and Vallorbes, Hotel Aubépine, from 5½ francs. Ballaigues is much more retired. Vevey being on the lake is lively, and there are plenty of excursions. Meiringew, on the Brunig railway, is a fascinating spot; Hotel Croix Blanche, from 5 francs.

Siena (Linguist).—The Italian spoken there is very pure, living is cheap, but you must set against that the heavy expense of moving a large family. As for gaiety, I do not think you would find much. The very little there is would be in the native society, to enter which you would have no chance without introductions. For gaiety you should go to Florence or Rome, where the English Colonies are so large. A short while since there was no English church at Siena, but there may be now. There is one at Perugia, at least there is a chaplain in the season, and service is held at one of the hotels.

Holiday in August (Nurse Mary).—Ten pounds is a very small sum for a fortnight's holiday in Switzerland on account of the expenses of the journey itself. Saxon Switzerland will not do. At Thun you can live for 6 francs a day, and also at Meiringen; this latter is well placed for small excursions. Then there is Grindlewald, quite among the mountains, but I prefer Meiringen, on account of its being on the Brunig Pass railway. Your board would come to a little over £4 each for fifteen days, counting tips, and your return journey, roughly speaking, to £5 10s. return. You have, therefore, no margin for excursions. How would you like the Belgian Ardennes? The country in the valley of the Meuse is lovely, but, of course, no mountains. You could live at Dinant for the same sum, and your return journey would only be £2. The difficulty of your friend and the table d'hote I see no way of overcoming unless you took lodgings, and those for a short time are more expensive. How would you like St. Servan, on the bay of St. Malo? Same hotel terms, journey £1 19s. Let me know when you decide on locality if I can help you further.

### For Reading to the Sick.

"THE first Day of the week."

O day of rest and gladness,
O day of joy and light,
O balm of care and sadness,
Most beautiful, most bright;
On thee the high and lowly,
Before the Eternal Throne,
Sing Holy, Holy, Holy,
To the great Three in One.

On thee, at the creation,
The light first had its birth;
On thee for our salvation
Christ rose from depths of earth:
On thee our Lord victorious
The Spirit sent from heaven;
And thus on thee most glorious
A triple light was given.

Thou art a cooling fountain
In life's dry deary sand;
From thee, like Pisgah's mountain,
We view our promised land;
A day of sweet reflection,
A day of holy love,
A day of resurrection
From earth to things above.

New graces ever gaining
From this our day of rest,
We reach the Rest remaining
To spirits of the blest.

—*W. Bright, D.D.*

"Sunday comes to us as a good gift from God. The return of Sunday is a knock at the door of our hearts by the hand of Him who is the very Light of the world. Sunday comes to us as an ordinance of the Christian Church with venerable authority. Sunday comes to us as absolutely needful refreshment for body, mind, and spirit. And I look upon Sunday as a good gift of God to us for our refreshment. . . . We are bound, if the gift of Sunday is a gift from God, to face the question of its observance for ourselves, to consider what shall be our own personal conduct on that day, to come to some decision as in the sight of God, as to what we had best do and not do. . . . Two points I want to press; first, Sunday, the Lord's Day, is for the refreshment of body, mind, and spirit—not of one only, not of two only, but all three; and secondly, Sunday, the Lord's Day, is for worship, and therein especially for Holy Communion."—*Heywood.*

"The Christian motive for observing the Lord's Day is the resurrection of Christ from the dead. That truth is to the Christian creed what the creation of the world out of nothing is to the Jewish. The Lord's Day marks the completed redemption, as the Sabbath had marked the completed creation. The resurrection is also the fundamental truth on which Christianity rests; and thus it is as much insisted on by the Christian apostles, as is God's creation of all things by the Jewish prophets."—*Liddon.*

This is the day of light;
Let there be light to-day;
O Day-spring, rise upon our night,
And chase its gloom away.
This is the day of rest:
Our failing strength renew:
On weary brain and troubled breast
Shed Thou Thy freshening dew.
This is the day of peace;
Thy peace our spirits fill;
Bid Thou the blasts of discord cease,
The waves of strife be still.
This is the day of prayer:
Let earth to Heav'n draw near;
Lift up our hearts to seek Thee there,
Come down to meet us here.
This is the first of days:
Send forth Thy quickening Breath,
And wake dead souls to love and praise
O Vanquisher of Death. Amen.

—*Ellerton.*

### Notes and Queries.

**THE Editor is always willing to answer in this column, without any fee, all reasonable questions, as soon as possible.**

**But the following rules must be carefully observed:—**

1. **Every communication must be accompanied by the name and address of the writer.**
2. **The question must always bear upon nursing, directly or indirectly.**

**If an answer is required by letter a fee of half-a-crown must be enclosed with the note containing the inquiry.**

*District Nursing.*

(149) 1. What are the definite duties of a district nurse in a rural parish? 2. Would a nurse who undertakes the work of a parish and surrounding hamlets—population 1,000, radius three miles—be expected to sit up by night with critical cases in the parish, at the same time to attend to the district work by day, and do her own housework and cooking? 3. Is a district nurse supposed to be "let out on hire" by the committee to the farmers of the parish for operations, &c.—to live at the house of patient by the week—still to attend to the work of the parish, and the money so earned to go to the "parish nursing fund?" 4. Can a midwife nurse refuse to take fever cases in the parish, where there is no doctor residing, even if the doctor in neighbouring town consents to take up the midwifery work at the time? It goes without saying that a midwife must expect a certain amount of night work, but I never heard before that a district nurse was expected to sit up by night with the sick in the district. 5. The remuneration being 17s. weekly (inclusive of everything), and two furnished rooms.—*Nurse Olivia.*

1. The duties should be defined in the contract of engagements. 2. Certainly not. 3. (As answer 1). 4. She ought to refuse, but she risks dismissal by doing so. 5. The scale of remuneration is much below the minimum required for a Queen's nurse.

*Obstinate Constipation.*

(150) Could you kindly suggest any permanent remedy for obstinate constipation? The usual methods seem simply to alleviate for a little time. I should be so grateful for your advice in the matter.—*A. F.*

We are always glad to help our correspondents, but it is impossible for us to prescribe for them. You must put yourself under the care of a medical man.

*Monthly Nurse's Fees.*

(151) Would you kindly tell me whether I can claim for laundry and travelling fare under the following circumstances? My terms were £10 10s. a month, 2s. 6d. weekly for laundry, and my fare. The lady said she had only before paid £8 8s., but would ask her husband. Subsequently she wrote saying that he had agreed to give me 2½ guineas weekly, for three weeks certain. Ultimately she asked me to stay another week. At the end of the time I was paid £10 10s. only, the gentleman refusing to pay me laundry and fare, as he had never promised to do so. Can I claim the 15s.?—*Helena.*

As the laundry and fare allowances were not mentioned in the letter, which partook of the nature of an agreement, we fear that you have no legal claim.

*South Africa.*

(152) I wrote the other day to the Army Nursing Reserve, being very anxious to go to South Africa. Their papers require you to have three years' training, and, unfortunately, I was trained at the Royal Southern Hospital, Liverpool, at the time when a certificate was granted at the end of twelve months, but I have had much and varied experience. Can you tell me what to do?—*Hope.*

Since your application the circumstances have somewhat altered. You might fill in the form, stating your case fully, and await the result.

*Nursing.*

(153) I am anxious to take up nursing in the East-end of London during the winter months. Can you tell me of any "home" where ladies are admitted as workers? I wish very much to go in for a hospital training, but cannot bind myself, as I must spend the holidays with my boy. I should be free from the end of September.—*E. H.*

Your best plan would be to join a "settlement," of which there are several in the East of London. The Canning Town Women's Settlement, 461, Barking Road, E., might suit you, as medical nursing is a speciality there. For others see the "Englishwoman's Year Book," but you would not become a "trained nurse" by these means.

*Hot Pack—Insomnia.*

(154) Would someone experienced in private nursing kindly tell me how she would give a "hot pack" in a private house? 2. Could some private nurse tell me of anything she has found successful when her patient has been very wakeful at night?—*M. S. C.*

1. Wring a blanket out of boiling water, lay it open upon a dry one spread ready on the bed, and place the patient upon it. Fold the wet blanket well round the trunk of the body, under as well as over the arms, and cover all with blankets to keep in the warmth. The bed should be protected with a mackintosh. 2. Gentle rubbing is excellent, but a nurse must study the idiosyncrasies of each patient in struggling with disorder that sometimes baffles the highest skill.

**Standard Books of Reference.**

"The Nursing Profession: How and Where to Train." 2s. net.
"The Nurses' Dictionary of Medical Terms." 2s.
"Burdett's Series of Nursing Text-Books." 1s. each.
"A Handbook for Nurses." (Illustrated.) 5s.
"Nursing: Its Theory and Practice." New Edition. 3s. 6d.
"Helps in Sickness and to Health." Fifteenth Thousand. 5s.

All these are published by THE SCIENTIFIC PRESS, Ltd., and may be obtained through any bookseller or direct from the publishers, 28 & 29, Southampton Street, London, W.C.

